# Two large-scale analyses of Ty1 LTR-retrotransposon *de novo* insertion events indicate that Ty1 targets nucleosomal DNA near the H2A/H2B interface

**DOI:** 10.1186/1759-8753-3-22

**Published:** 2012-12-17

**Authors:** Antoine Bridier-Nahmias, Pascale Lesage

**Affiliations:** 1 CNRS/P7 UMR7212, INSERMU 944, Laboratoire Pathologie et Virologie Moléculaire Institut Universitaire d'Hématologie, 1 avenue Claude Vellefaux, F75010, Paris, France; 2 Inserm U944, Institut Universitaire d’Hématologie, Hôpital St Louis, Paris, France; 3 Université Paris Diderot, Sorbonne Paris Cité, Hôpital St Louis, Paris, France

**Keywords:** LTR retrotransposon, Ty1, Selective integration, Large-scale analysis

## Abstract

**Background:**

Over the years, a number of reports have revealed that Ty1 integration occurs in a 1-kb window upstream of Pol III-transcribed genes with an approximate 80-bp periodicity between each integration hotspot and that this targeting requires active Pol III transcription at the site of integration. However, the molecular bases of Ty1 targeting are still not understood.

**Findings:**

The publications by Baller et al. and Mularoni et al. in the April issue of Genome Res. report the first high-throughput sequencing analysis of Ty1 de novo insertion events. Their observations converge to the same conclusion, that Ty1 targets a specific surface of the nucleosome at he H2A/H2B interface.

**Conclusion:**

This discovery is important, and should help identifying factor(s) involved in Ty1 targeting. Recent data on transposable elements and retroviruses integration site choice obtained by large-scale analyses indicate that transcription and chromatin structure play an important role in this process. The studies reported in this commentary add a new evidence of the importance of chromatin in integration selectivity that should be of interest for everyone interested in transposable elements integration.

## Findings

Retrotransposons are major components of eukaryotic genomes. They represent, for example, half of the human genome, up to 80% of some plant's genomes and 3% of the compact genome of Yeast *S. cerevisiae.* They have a central role in shaping genomes, and have been shown to be a powerful force of evolution and to play a positive role in long-term adaptation. However, they can also be deleterious in the short-term, since their integration into the host genome can inactivate or deregulate gene expression or even induce large chromosomal rearrangements by homologous recombination of distant copies. LTR-retrotransposons are structurally and functionally related to retroviruses but their life cycle is exclusively intracellular since they do not encode an envelope glycoprotein. They replicate by reverse transcribing their RNA into cDNA, which is ultimately integrated into the host genome by the element-encoded integrase (IN). The non-random distribution of LTR-retrotransposons and retroviruses into genomes suggests that these elements actively select their integration sites (for review, [1]).

**Figure 1:**
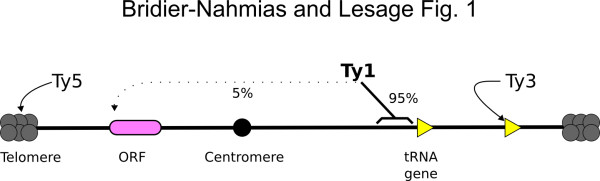
Yeast chromosome with Ty1, 3, 5 integration-targeted regions.

For the past 20 years, studies on Ty3 and Ty5 LTR-retrotransposons of *S. cerevisiae* have led to better understand the molecular bases of their targeted integration (Figure 1). It has been established that an interaction between Ty3 IN and the Brf1 subunit of TFIIIB is sufficient to target Ty3 integration to Pol III transcription initiation sites *in vitro*[2]. Likewise, *in vivo*, Ty5 preferential integration into silent telomeric heterochromatin depends on the interaction between Ty5 IN and the Sir4 protein, a structural component of silent chromatin [3]. In the distant Yeast *S. pombe*, interaction between Tf1 IN and the Atf1 transcription factor plays a direct and specific role in targeting Tf1 integration in the *fbp1* gene promoter [4]. These studies have converged on a common targeting mechanism, based on tethering of integration complexes to the cell genome through interaction between IN and cellular proteins bound at favored insertion sites. It is noteworthy that this model may also account for the selectivity of the integration of retroviruses, since HIV-1 integration in active transcription units relies on the interaction of its IN with the LEDGF/p75 transcription factor (reviewed in [5]).

Discovered in 1979 [6], Ty1 is the most abundant and active LTR-retrotransposon in *S. cerevisiae*. It was first noticed in 1982 that Ty1 insertions are located adjacent to several tRNA genes [7]. Over the years, a number of reports have found that Ty1 integration occurs in a 1-kb window upstream of Pol III-transcribed genes with an approximate 80-bp periodicity between each integration hotspot and that this targeting requires active Pol III transcription at the site of integration (Figure 1) [8-10]. However, despite 30 years of active research, the molecular bases of Ty1 targeting are still not understood. Thus, the recent articles of Baller *et al.* and Mularoni *et al.* are an important advance for understanding the selection of Ty1 integration sites, by showing that Ty1 targets a specific surface of the nucleosome [11,12].

**Figure 2:**
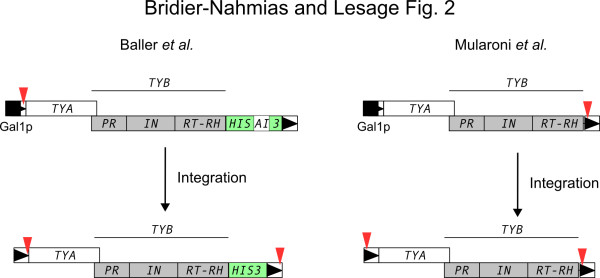
**Strategies used to recover insertions: red triangles represent the tag sequences transferred from one LTR to another during retrotransposition.** In Baller *et al.*, the tag is a 6 nucleotide substitution, in Mularoni *et al.*, the tag is a 25 bp synthetic DNA.

Both studies used a deep-sequencing approach to get insights into Ty1 integration selectivity. To discriminate *de novo* insertion events from resident Ty1 elements, both used a short tag sequence introduced in one LTR of a galactose-inducible donor Ty1 element, such that after a complete retrotransposition cycle the tag would be recovered in both LTRs (Figure 2), and this short tag was used to specifically sequence newly transposed sequences. In the Mularoni *et al.*'s study, sequences were generated by an Illumina GAII apparatus. Of a total of 7,990,112 reads, 1,154,281 were characterized as non-redundant Ty1 insertions. Baller *et al.* used a donor Ty1 element, which contained the *his3*AI reporter construct conferring histidine prototrophy to the cells after Ty1 retrotransposition, and recovered only His^+^ insertions. A single 454 run produced from 13,000 to 111,000 reads. While Mularoni *et al.* analyzed 10- to 100-fold more integration events than Baller *et al.,* the latter analyzed integration profiles in diploid and haploid wild-type cells and in a panel of mutants affecting DNA-related processes known to increase integration frequency (*rrm3Δ, rtt109Δ, hos2Δ*) and integration in coding sequences and transcription units (*rad6*Δ). *RTT109* and *HOS2* encode histone-modifying enzymes, while *RRM3* encodes a helicase and *RAD6* an ubiquitin-conjugating enzyme.

Both reports describe the same general insertion profile pattern in wild-type cells. Independently of cell ploidy, a vast majority (~90%) of insertions were observed as predicted in the 5' region of class III genes. However, Ty1 did not target class III genes equally. All but two tRNA genes, tE(UUC)C and tI(AAU)L1, received many insertions. Other class III genes, such as *SNR6, RPR1, SNR52, SCR1* and the repeated locus *RDN5* received insertions as well. However, two class III loci of unknown function, *RNA170* and *ZOD1*, were not targeted, probably because the Pol III transcription machinery was not efficiently recruited at these loci. Insertions in Pol II-transcribed genes were rare, representing about 5% of total events. Most of them occurred near a Pol III gene, with a strong preference for the region closest to the class III gene. Considering ORFs which are more distant to tRNA genes (~5 kb), the few recovered insertions occurred at the gene 5' end. Although insertions into mitochondrial sequences (mtDNA) were reported by Mularoni *et al.*, they were not detected by Baller *et al.*, probably because of a smaller dataset size or because those insertions did not confer histidine prototrophy and were, consequently, not selected for sequencing. Mularoni *et al.* suggest that these insertions might come from shattered mitochondria and could occur in the nucleus or even in the cytoplasm.

**Figure 3:**
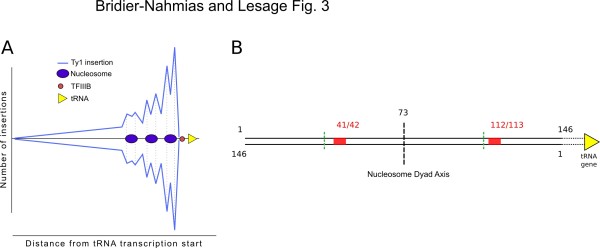
**(A) Plot of Ty1 insertions upstream of tRNAs.** The blue curve above the midline represents Ty1 insertions in tandem with the tRNAs, that below the midline represents elements inserted in inverted orientation. (**B**) Scheme of Ty1 integration in nucleosomal DNA. The black lines represent nucleosomal DNA from base 1 to base 146 (5' to 3' orientation), the red boxes are the hotspots of integration with the coordinates of the attacked dinucleotides indicated above. The green broken lines indicate the expected integration hotspots symmetrically disposed around the dyad axis and distant of 73 bp. We can see here the "right shift" of the observed integration hotspots towards the tRNA gene in regard to the dyad axis of symmetry.

The novel and most striking observation of both studies is that Ty1 integration is positively correlated with nucleosome occupancy. An important role for chromatin in the selection process of insertion sites was already suspected after the discovery of an intriguing periodicity of ~80 bp between each integration hotspots that relied on the ATP-dependent chromatin remodeling factor Isw2 [10]. By comparing their deep-sequencing results with genome-wide nucleosome positioning data sets, they have discovered two hotspots per nucleosome, separated by about 70 bp. These observations were made for the first three nucleosomes directly upstream of a class III gene and the integration events were aligned with the nucleosome H2A/H2B interface (Figure 3).

In *hos2Δ* and *rtt109Δ* mutant strains analyzed by Baller *et al.*, the pattern of Ty1 insertion events was not significantly different from that in wild-type cells. In contrast, integration events in verified ORFs increased significantly in *rrm3Δ* and *rad6Δ* mutant strains (by two- and three-fold, respectively), although the integration pattern upstream of tRNA genes was unmodified, leading to the conclusion that the determining factors for specific nucleosomal targeting upstream of class III genes were not affected in these mutants.

High-throughput sequencing of insertion events has provided a saturated profile of target activity for several retrotransposons and contributed to better understand their integration preferences. For example, integration of the LTR retrotransposon of S*. pombe* Tf1 has been shown to be strongly biased for Pol II promoters with a clear preference for stress-induced promoters [13], and another report, in which 10,000 events have been analyzed, confirms all previous *in vitro* evidences on Ty3 integration at Pol III sites [14]. A high-throughput sequencing strategy has also been largely used to map the insertions profiles of different retroviruses (for review [15]). The reports of Mularoni *et al.* and Baller *et al.* reveal that Ty1 integration upstream of class III genes is strongly correlated with the chromatin structure at these loci and preferentially targets a specific nucleosomal DNA segment. Interestingly, the preference for nucleosome-rich regions is not a conserved feature of retroelements since it has been shown that elements such as Ty5 and Hermes (when expressed in yeast) prefer nucleosome-free regions [16,17].

Although these two studies clearly contribute to better understanding of Ty1 targeting, they do not characterize whether a specific nucleosomal DNA conformation, a specific histone modification, or a nucleosome-bound factor enriched at sites of Pol III transcription, determine Ty1 preferred target sites, nor do they elucidate the role of RNA polymerase III and its co-factors in Ty1 targeting. Thus, further work is required to completely decipher the molecular bases of Ty1 targeting.

## Abbreviations

H2A: Histone protein H2A; H2B: Histone protein H2B; His+: Histidine prototrophy; HIV-1: Human Immunodeficience Virus 1; IN: Integrase; LTR: Long Terminal Repeat; ORF: Open Reading Frame; Pol III: RNA Polymerase III; Ty: Transposon in yeast.

## Competing interests

The authors declare that they have no competing interests.

## Authors’ contributions

AB-N, wrote the manuscript PL, supervised the final manuscript. All authors read and approved the final manuscript.
